# Is There Bias in Virtual Interviews for Emergency Medicine Residency?

**DOI:** 10.7759/cureus.109039

**Published:** 2026-05-17

**Authors:** Matthew W Morgan, Nathan D Baggett, Katrina M Greenwalt, Graci M Gorman

**Affiliations:** 1 Emergency Medicine, Regions Hospital, St. Paul, USA

**Keywords:** bias, emergency medicine, residency application process, residency interview, virtual interview

## Abstract

Background

Since the COVID-19 pandemic, residency programs have increasingly relied on virtual interviews. The impact of the interview format on interview scoring remains uncertain. As more programs offer both virtual and in-person interviews, understanding how these formats impact evaluations is increasingly essential. This study aimed to assess bias in applicant interview scores and determine whether the format (virtual vs. in-person) is associated with differences in interview scores.

Methodology

Data were collected on two years’ worth (2023-2025) of interviewed applicants retrospectively through the Electronic Residency Application System and anonymized. After assessing baseline characteristics between virtual and in-person applicants, a linear regression analysis was performed to assess associations between several predictors and interview score.

Results

A total of 241 applicants were interviewed over the two years that data were collected. Of those, 130 (53.9%) were interviewed virtually and 111 (46.1%) in-person. Assessed baseline characteristics were similar between groups, other than the frequency of median standardized letter of evaluation (SLOE) scores greater than two and state of residence. Linear regression accounted for 27.3% of the variance in interview scores (indicating moderate explanatory power). Golden Humanism and Alpha Omega society membership, as well as female gender and SLOE scores, were positively correlated with interview scores. Interview format (in-person vs. virtual) was not a significant predictor of interview score.

Conclusions

There was no difference in interview scores between in-person and virtual interviewees when controlled for other factors.

## Introduction

Over the past four years, residency programs have increasingly relied on virtual interviews, a shift primarily driven by the Coalition for Physician Accountability (CoPA). This organization, which includes the Association of American Medical Colleges (AAMC), the Accreditation Council for Graduate Medical Education (ACGME), and the American Medical Association (AMA), recommended virtual interviews during the COVID-19 pandemic to prioritize safety. However, since 2021, little formal guidance has been provided, leading some programs to reintroduce in-person interviews alongside virtual options.

Many studies have examined applicants’ perspectives on these interview formats. According to Domingo et al., most applicants found virtual interviews effective, citing advantages such as cost savings, time efficiency, reduced travel burden, and a lower carbon footprint. While some drawbacks were noted, including time zone differences, the availability of suitable interview environments, and reliable internet access, 84% of participants still expressed a preference for maintaining virtual interviews in the future. The study found no significant differences in responses based on gender, geographic location, or race [1].

In contrast, Seifi et al.found that while both medical students and residents preferred in-person interviews, they still supported keeping virtual interviews [2]. This suggests a more nuanced perspective, where some individuals value the interpersonal aspects of in-person interviews, while others prioritize the convenience of virtual interactions.

Other research has focused on potential biases and strategies to mitigate them in virtual interviews. Otugo et al. outlined several methods to reduce bias, including standardizing virtual backgrounds, providing audio recordings for name pronunciation, conducting technology checks, and offering implicit bias training for interviewers [3].

While prior research has focused largely on applicant satisfaction and perceptions, relatively fewer studies have examined whether interview format affects objective outcomes such as interview scoring. Das et al. demonstrated that although perceptions of virtual interviews differed, match outcomes were not significantly impacted by interview format [4]. As more programs offer both virtual and in-person interviews, understanding how these formats impact evaluations is increasingly essential.

During the 2023-2024 and 2024-2025 residency interview cycles, our program offered applicants both an in-person and virtual interview option. This study aimed to assess bias in applicant interview scores between formats (virtual vs. in-person). After one year of data collection, the data to that point were presented as an abstract at the ACEP Annual Scientific Assembly on September 30, 2024. The present study incorporates an additional year’s data.

## Materials and methods

Study design

The present study is a single-center, retrospective, observational cohort study that compares interview scores based on interview format (in-person vs. virtual) using linear regression to assess for confounders. Interview scores were scores from 1-10 (10 being a high score) given by each of the applicants’ three interviewers.

Inclusion and exclusion criteria

All applicants’ data for two years running (2023-2025) were included for analysis. No applicants’ data were excluded.

Data collection

Data were collected on two years’ worth (2023-2025) of interviewed applicants retrospectively through the Electronic Residency Application Service and anonymized. Data collected included interview format, interview score, Step 2 score, standardized letter of evaluation (SLOE) scores, gender, underrepresented in medicine status, Alpha Omega Alpha (AOA) status, Golden Humanism Honor Society (GHHS) status, and state/regions of residence. Given that the data were anonymized, this study was deemed exempt from formal IRB review.

Statistical analysis

Baseline characteristics were analyzed for similarity between groups (virtual interviews vs. in-person). Step 2 scores were normally distributed, and a two-sample t-test was used to assess similarity between groups. The percentage of median eSLOE scores >2 was calculated, and an odds ratio was obtained. The electronic SLOE (eSLOE is scored 1-3, with 3 being the top score. Female/male, underrepresented in medicine, AOA membership, and GHHS membership were compared using a chi-square test of independence. State of residence was categorized as Minnesota, the Midwest, and other. These frequencies were compared using the chi-square test of independence. Given that baseline characteristics were similar between groups (aside from median eSLOEs and state of residence), we directly compared interview scores to minimize the potential for type 1 error compared to matching techniques. A linear regression analysis was then performed to assess associations between the above predictors and interview score.

## Results

A total of 241 applicants were included across two residency application cycles, of whom 130 (53.9%) interviewed virtually and 111 (46.1%) interviewed in-person. Baseline applicant characteristics were compared between interview format groups (Table 1). Step 2 scores were similar between applicants who interviewed virtually and those who interviewed in-person (251 vs. 248, p = 0.093). There were no statistically significant differences between groups in gender distribution, underrepresented in medicine status, AOA membership, or GHHS membership (all p > 0.05).

**Table 1:** Baseline characteristics. Data are presented as mean values or frequencies. Continuous variables were compared using independent-samples t-tests, and categorical variables using chi-square or Fisher’s exact tests. Test values are reported alongside corresponding p-values AOA: Alpha Omega Alpha; GHHS: Golden Humanism Honor Society; MN: Minnesota; MW: Midwest; SLOE: standardized letter of evaluation

		In-person (n = 111)	Virtual (n = 130)	Test	Unadjusted p-value
Step 2 CK score		248	251	t = 1.68	0.093
Median SLOE >2 frequency, N (%)		62 (56%)	101 (78%)	Fisher’s exact test	<0.001
Female applicant frequency, N (%)		67 (60.4%)	74 (56.9%)	Chi-square = 0.29	0.589
Underrepresented minority frequency, N (%)		11 (9.9%)	19 (14.6%)	Chi-square = 1.22	0.270
AOA frequency, N (%)		14 (12.6%)	13 (10.0%)	Chi-square = 0.41	0.521
GHHS frequency, N (%)		16 (14.4%)	20 (15.4%)	Chi-square = 0.04	0.833
State of residence frequency, N (%)	MN	86 (77.9%)	36 (27.7%)	Chi-square = 49.10	<0.001
	MW	24 (21.6%)	67 (51.5%)		
	Other	1 (0.9%)	27 (20.8%)		

However, significant differences were observed in SLOE performance and geographic distribution. A greater proportion of applicants in the virtual interview group had an SLOE score of greater than 2 compared to the in-person group (101 vs. 62, p < 0.001). Additionally, applicants who interviewed in person were more likely to be from Minnesota, whereas virtual interviewees were more likely to be from outside the region (p < 0.001).

A multivariable linear regression model was constructed to evaluate the association between interview format and interview scores while adjusting for applicant characteristics (Table 2). Linear regression accounted for 27.3% of the variance in interview scores, indicating moderate explanatory power.

**Table 2:** Multivariable linear regression predicting the interview score. Beta coefficients represent the estimated change in interview score associated with each variable. Confidence intervals and adjusted p-values are reported. P-values are derived from the multivariable linear regression model.

	Beta coefficient	95 % confidence interval	Adjusted p-value
Interview format	0.15	-0.16–0.46	0.35
Underrepresented minority	0.23	-0.17–0.63	0.27
Golden Humanism Honor Society	0.43	0.05–0.81	0.03
Alpha Omega Alpha Honor Society	0.48	0.04–0.92	0.03
Female gender	0.46	0.18–0.73	0.001
State of residence	0.13	-0.09–0.36	0.25
SLOE	0.84	0.58–1.09	<0.001
Step 2 score	0.00	-0.01–0.01	0.76

SLOE scores were positively associated with interview scores (B = 0.84, ρ < 0.01), indicating that those with median SLOE scores of greater than 2 scored 0.84 points higher (on a 10-point scale). AOA membership was also positively associated with interview scores, as was female gender and GHHS membership. Interview format (in-person vs. virtual) was not a significant predictor of interview score, nor were any of the other independent variables (underrepresented minority status, state residence, or Step 2 score).

## Discussion

The present study found that the interview format, whether virtual or in-person, did not significantly impact the scores applicants received from interviewers after controlling for relevant factors. This suggests that offering applicants a choice of interview format does not inherently disadvantage those choosing to interview virtually. Given the applicants’ self-selected interview format, it is possible, though felt less likely, that there are other significant unmeasured differences unrelated to interview format. This aligns with an expanding body of evidence demonstrating that virtual interviews are a viable and equitable alternative to in-person formats. Several prior studies have examined objective outcomes related to interview format. Lamberton et al. found no significant difference in faculty scoring between virtual and in-person interviewees in a general surgery residency cohort, suggesting that interviewer assessments remain consistent across modalities [5]. Similarly, a systematic review by Daniel et al. concluded that there is limited evidence to support meaningful differences in applicant evaluation or ranking based on interview format, although objective scoring data were limited in the studies contained in their review [6]. Another study looking at fellowship interviews in a surgical program found no significant difference in evaluation outcomes between virtual and in-person interviews [7].

Other factors influenced interview scores in the present study, including SLOE ratings, AOA membership, GHHS membership, and female gender. The model demonstrated moderate explanatory power, which suggests that additional, unmeasured factors contribute to interview performance. This is an expected finding, as interviews are intended to assess qualities such as interpersonal skills, professionalism, and personality, factors that other application components may not fully capture. Prior work has emphasized the role of interviews in evaluating these dimensions of applicant performance [8].

Concerns about bias in an entirely virtual residency selection process have been explored in the literature. Potential sources of bias include differences in access to technology, interview environment, and limitations in assessing nonverbal communication. Fuchs and Youmans highlighted that while some of these new forms of biases may be introduced, other traditional inequities related to travel cost and geographic constraint may be mitigated by virtual interviews [9]. Another study suggested that structured interview formats and interview training could be used to reduce bias in interview settings [10]. Other work has suggested that virtual interviews may improve accessibility while requiring careful standardization to maintain equity [11].

Applicant and program perceptions of interview formats provide additional context. Domingo et al. and Serif et al. demonstrated that applicants generally view virtual interviews favorably due to reduced cost and logistical burden. They may still prefer in-person interactions for assessing program culture [1,2]. Program directors have expressed mixed perspectives, with some concerned about limitations in evaluating interpersonal fit in virtual settings [12]. More recent studies suggest that hybrid approaches, including optional in-person visits or second look opportunities, are increasingly common. One study found that optional in-person visits may enhance applicant experience without significantly influencing ranking decisions [13]. However, other studies have raised concerns that such models may introduce unintended bias if participation varies among applicants [14,15].

The findings of the current study add to the current body of literature that suggests that virtual interviews can be incorporated into residency selection processes without compromising fairness in applicant evaluation. As recruitment strategies continue to evolve, careful attention should be paid to newer formats, such as hybrid models do not reintroduce bias with the in-person components.

This study has some limitations. As a single-institution analysis, its findings may not be generalizable to all programs. Additionally, while the model explained a moderate portion of the variance in interview scores, other important factors likely play a role. Further research is needed to assess potential biases in virtual interviews and to ensure that applicants who opt for this format are evaluated equitably.

## Conclusions

The interview format was not associated with differences in residency applicant interview scores in this study. These findings suggest that virtual interviews may not introduce bias into the application process. Further study is needed to better understand how the interview format may influence residency selection outcomes.
